# Understanding adolescent males’ poor mental health and health-compromising behaviours: A factor analysis model on Swedish school-based data

**DOI:** 10.1177/1403494820974555

**Published:** 2020-12-15

**Authors:** Johanna Haraldsson, Ronnie Pingel, Lena Nordgren, Ylva Tindberg, Per Kristiansson

**Affiliations:** 1 Department of Public Health and Caring Sciences, Uppsala University, Sweden; 2 Centre for Clinical Research, Sörmland/Uppsala University, Sweden; 3 Department of Statistics, Uppsala University, Sweden; 4 Department of Women’s and Children’s Health, Uppsala University, Sweden

**Keywords:** Adolescent, mental health, risk taking, factor analysis, statistical, cross-sectional studies, male

## Abstract

Aim: The aim was to develop a factor model of the clustering of poor mental-health symptoms and health-compromising behaviours (HCBs) in adolescent males. Methods: The study was based on two cross-sectional school-based Swedish surveys in 2011 (response rate 80%, *N*=2823) and 2014 (response rate 85%, *N*=2358), both of which comprised questionnaires from males aged 15–16 and 17–18 years. A factor model was developed by exploratory factor analysis on the 2011 survey and validated by confirmatory factor analysis on the 2014 survey. Results: Four aspects of poor mental health and HCBs emerged in the exploratory factor analysis: (a) deviancy as a tendency to substance use and delinquency, (b) unsafety as an inclination towards feelings of unsafety in different environments, (c) gloominess as a tendency towards pessimism and feeling unwell and (d) pain as an inclination to experience physical pain. The model was validated with good model fit. Age did not affect the model structure, but older adolescent males were more influenced by deviancy and gloominess and less by unsafety compared to their younger peers. **Conclusions: Separating symptoms of poor mental health and HCBs into four areas – deviancy, unsafety, gloominess and pain – brings new perspectives to the understanding of adolescent males’ health. To the best of our knowledge, our factor model is the first to include unsafety and pain in this context. Whenever a comprehensive approach to the health of adolescent males is needed in the clinic or in the field of public health, this factor model may provide guidance.**

## Introduction

Adolescent males have been disadvantaged in the past decades’ global health improvements [1]. Although many deaths are preventable [2], the overall mortality rate in males aged 15–19 years is twice that of females their age [1,3]. Much of the morbidity and mortality derive from poor mental health and health-compromising behaviours (HCBs) [2,4] that can persist into adulthood and contribute to the public-health burden [2].

Symptoms of poor mental health have increased in adolescent males in the Nordic countries since the 80s, by far most in Sweden [5]. Moreover, poor mental health is associated with somatic symptoms such as headache and stomach ache [6,7], and with engagement in multiple HCBs [8], that is, behaviours that can impair the adolescent’s health or development into a well-functioning adult.

Many adolescents engage in a small number of HCBs as a part of normal development [9,10]. However, in some individuals, HCBs tend to cluster [8,10–14]. According to *The Problem Behaviour Theory* by Jessor [11], the clustering of HCBs is caused by an underlying factor, reflecting an inclination for engagement in various HCBs [11,15].

Assuming that clustering can be explained by underlying factors, we hypothesised that the aforementioned co-occurrence of poor mental health, somatic symptoms and HCBs is related to one or more such factors reflecting a tendency towards both poor health and engagement in HCBs. However, we have found no factor model that comprehensibly includes these perspectives, that is, comprises symptoms of poor mental health, somatic symptoms, substance use (e.g. tobacco, alcohol and other drugs), delinquency and sexual risks while taking their covariation into account. Besides, different models might be required for different age groups [12,16]. Hence, it is unknown if poor mental health, somatic symptoms and HCBs share the same underlying factors or if there are age-related differences. A factor model that conceptualises the major categories of adolescent public health and their relationships can lend support to public-health professionals [2].

## Aim

The aim of this study was to develop a factor model of the clustering of poor mental-health symptoms, somatic symptoms and HCBs in adolescent males using exploratory factor analysis (EFA) and confirmatory factor analysis (CFA).

## Methods

### Study design and setting

A descriptive cross-sectional study design was used with data from two independent samples from different occasions. A factor model was developed from the first sample using EFA, and validated in the second sample using CFA.

Data were obtained from a triannual school-based survey, Life and Health in Youth, conducted by the Centre for Public Health and the Centre for Clinical Research Sörmland at Region Sörmland, Sweden. The survey targets all schools in the region of Sörmland, a socio-economically representative area of Sweden [3].

Data were collected from male students in year 9 of compulsory school (Y9; 15–16 years old) and year 2 of upper secondary school (Y2U; 17–18 years old) in 2011 and 2014. The setting, selection of participants and data collection were identical in the two surveys, with one exception: in 2011, Y2U students attending schools outside the region received their questionnaires by mail for completion at home, whereas in 2014, no questionnaires were mailed.

### Data collection

In March 2011 and 2014, the students completed the questionnaires anonymously in the classroom during school hours [17]. School employees handed out, collected and returned the questionnaires to the Centre for Clinical Research, Sörmland. Absent students had a second opportunity to participate within two weeks. Beforehand, students and parents were informed in writing that participation was voluntary, and a completed questionnaire was therefore regarded as the student’s informed consent. To protect the participants’ identities, no formal written informed consent forms were used. Furthermore, no parental approval is needed in Sweden for participants aged ⩾15 years [18]. The study design was approved by the Regional Ethical Review Board, Stockholm (Dnr 2014/1955-32, 2017/709-32).

### Study population

In 2011, the response rate in the 62 (of 65) participating schools was 80% (84% in Y9; 77% in Y2U; Supplemental Figure S1). Of the 296 mailed questionnaires, 100 (34%) were returned completed. All questionnaires from male students were used (*N*=2823; Y9=1437, Y2U=1386).

In 2014, the response rate in the 64 (of 68) participating schools was 84% (86% in Y9; 84% in Y2U; Supplemental Figure S2). Six questionnaires were deemed unreliable and discarded, yielding 2358 usable questionnaires from male students (Y9=1139, Y2U=1219). Characteristics are presented in Table I.

**Table I.** Characteristics of the adolescent males in the study populations in the Life and Health in Youth survey 2011 and 2014.

Characteristics	2011		2014	
	Total	Missing	Total	Missing
	*n* (%)	*n* (%)	*n* (%)	*n* (%)
Male	2823 (100.0)	–	2358 (100.0)	–
*Grade*:				–
Y9^[Table-fn table-fn1-1403494820974555]^	1437 (50.9)	–	1139 (48.3)	
Y2U	1386 (49.1)	–	1219 (51.7)	
*Parents’ country of birth*^a^:		57 (2.0)		116 (4.9)
Sweden	2317 (83.8)		1825 (81.4)	
Other country in Europe	143 (5.2)		130 (5.8)	
Country outside Europe	306 (11.1)		287 (12.8)	
*Parents’ occupation*:		64 (2.3)		183 (7.8)
Both parents work or study	2178 (78.9)		1782 (81.9)	
One parent unemployed or on sick leave	474 (17.2)		343 (15.8)	
Both parents unemployed or on sick leave	107 (3.9)		50 (2.3)	
*Family situation*:		–		–
Living with both parents	1646 (58.3)		1370 (58.1)	
Living with separated parents	965 (34.2)		834 (35.4)	
Other	212 (7.55)		154 (6.5)	
*Chronic diseases and disabilities*:				
Somatic chronic disease^b^	433 (15.7)	57 (2.0)	377 (16.3)	41 (1.7)
Physical disability^c^	318 (11.8)	127 (4.5)	271 (12.0)	91 (3.9)
Dyslexia	321 (11.9)	132 (4.7)	293 (13.4)	177 (7.5)
ADHD and autism spectrum disorders	147 (5.5)	139 (4.9)	193 (8.7)	149 (6.3)

a If the parents were born in different countries, the category pertains to the country geographically closest to Sweden, in accordance with the recommendations of Statistics Sweden.

b Including diabetes mellitus, epilepsy, inflammatory bowel disease, asthma and severe allergy (mild allergy was not classified as a chronic disease). Available response options were ‘no’, ‘yes, mild’ and ‘yes, severe’.

c Including impaired vision (not correctable with glasses), hearing or mobility.

Y9: year 9 compulsory school; Y2U: year 2 upper secondary school; ADHD: attention-deficit/hyperactivity disorder.

### Questionnaires

The questionnaires differed slightly between surveys and school years. Each questionnaire comprised nearly 90 questions (87 in Y9 2011, 89 in Y2U 2011, 86 in Y9 2014 and 87 in Y2U 2014) [17], concerning sociodemographic background, somatic, mental and sexual health, school, spare time, HCBs and health care. The questionnaires were not validated but had been used previously [19,20].

### Item selection and description

Questions were selected that were worded equally in all four questionnaires and potentially measured an underlying factor (*n*=51). An underlying factor is a real but unobserved trait which causes the correlations between the manifest variables [21]. Four binary items, six highly correlated items and nine items with consistently low factor loadings were excluded. Finally, 32 items were used in the EFA.

The included items (Tables II and III) were ordinal, with response options varying from three- to seven-point scales. Their phrasing, response options and item constructions were identical in 2011 and 2014 (Tables II and III). For each item, the response option with the lowest potential negative health impact was coded as zero.

**Table II.** Dichotomised frequencies and proportions of symptoms, negative experiences and health-compromising behaviours reported in the 32 analysed items included in the exploratory factor analysis in the 2011 survey.

No.	The items, verbatim translated from the questionnaire^a^	All selectable response options^b^	All students,^c^ *n* (%)^d^	Y9, *n* (%)^d^	Y2U, *n* (%)^d^	Missing,*n* (%)	Difference, Y9–Y2U, *p*-value^e^
	Male students		2823 (100)	1437 (50.9)	1386 (49.1)		
	**School**						
1	Do you enjoy school?	A great deal, Quite a bit, Somewhat | *A little bit, Not at all*	132 (4.74)	76 (5.39)	56 (4.08)	41 (1.45)	0.106
2	Do you regularly skip class?	No, Once a semester, Once a month, 2 to 3 times a month| *Once a week, Twice a week or more*	239 (8.66)	86 (6.19)	153 (11.2)	63 (2.23)	<0.001
	**Spare time**						
3	Are you satisfied with your spare time?	Very satisfied, Rather satisfied, Neither satisfied nor unsatisfied | *Rather unsatisfied, Very unsatisfied*	126 (4.55)	47 (3.34)	79 (5.78)	51 (1.81)	0.002
4	In your spare time, how many times a week do you exercise at least 30 minutes vigorously enough to make you sweat?	Every day, 4 to 6 times a week, 2 to 3 times a week, Once a week | *1 to 3 times a month, Less than once a month, Never*	633 (22.9)	287 (20.6)	346 (25.4)	63 (2.23)	0.003
	**Somatic health**						
5	How is your general health?	Excellent, Good, Fair | *Poor, Very poor*	89 (3.17)	41 (2.87)	48 (3.48)	18 (0.64)	0.357
6	How often do you brush your teeth in an ordinary day?	3 times a day or more, 2 times a day | *Once a day, Never*	651 (23.5)	283 (20.0)	368 (27.0)	47 (1.66)	<0.001
	During the last three months, how often have you had. . .						
7	. . .headache (not counting migraine)?	Seldom or never, Once a month, Once a week | *More than once a week, Almost every day*	197 (7.21)	89 (6.42)	108 (8.02)	90 (3.19)	0.104
8	. . .stomach ache?	Seldom or never, Once a month, Once a week | *More than once a week, Almost every day*	125 (4.64)	64 (4.65)	61 (4.62)	128 (4.53)	0.974
9	. . .pain in your neck or shoulders?	Seldom or never, Once a month, Once a week | *More than once a week, Almost every day*	226 (8.42)	103 (7.53)	123 (9.34)	138 (4.89)	0.091
10	. . .pain in your back or hips?	Seldom or never, Once a month, Once a week | *More than once a week, Almost every day*	219 (8.19)	112 (8.20)	107 (8.17)	148 (5.24)	0.981
	**Mental health**						
11	Do you enjoy life right now?	A great deal, Quite a bit | *A little bit, Not at all*	180 (6.56)	65 (4.67)	115 (8.51)	81 (2.87)	<0.001
	During the last three months, how often . . .						
12	. . . have you been concerned or worried?	Seldom or never, Once a month, Once a week | *More than once a week, Almost every day*	399 (14.6)	175 (12.6)	224 (16.6)	86 (3.05)	0.003
13	. . . have you experienced low mood?	Seldom or never, Once a month, Once a week | *More than once a week, Almost every day*	349 (12.8)	135 (9.68)	214 (15.9)	87 (3.08)	<0.001
14	. . . have you been stressed?	Seldom or never, Once a month, Once a week | *More than once a week, Almost every day*	933 (33.7)	427 (30.3)	506 (37.3)	58 (2.05)	<0.001
15	. . .did you feel cheerful?	*Seldom or never, Once a month, Once a week |* More than once a week, Almost every day	239 (8.63)	100 (7.09)	139 (10.2)	53 (1.88)	0.003
16	. . . did you have difficulties getting into sleep?	Seldom or never, Once a month, Once a week | *More than once a week, Almost every day*	540 (19.8)	232 (16.7)	308 (23.0)	95 (3.37)	<0.001
17	During the last 12 months, how often have you tried to cut or injure yourself in any way?	Never | *Once, 2 to 5 times, More than 5 times*	158 (5.89)	71 (5.20)	87 (6.61)	141 (4.99)	0.120
	**Substance use**						
18	Do you smoke?	No, Have tried, Have stopped | *Sometimes, Daily.*	551 (19.7)	193 (13.6)	358 (26.1)	29 (1.03)	<0.001
19	During the last 12 months, how often have you been drinking alcohol?	Never, Once, A few times every six months | *1 to 3 times a month, 1 to 2 times a week, More than 2 times a week*	871 (31.5)	246 (17.5)	625 (46.0)	58 (2.05)	<0.001
20	How many times have you ever used illegal drugs?	Never | *Once, Twice or more*	347 (12.6)	98 (7.01)	249 (18.2)	59 (2.09)	<0.001
	**Delinquency**						
	Have you ever. . .						
21	. . .taken goods from a shop without paying?	Never, Once, 2 to 5 times | *More than 5 times*	300 (10.9)	140 (10.0)	160 (11.8)	59 (2.09)	0.120
22	. . .broken into a house or a car?	Never | *Once, 2 to 5 times, More than 5 times*	409 (14.9)	169 (12.1)	240 (17.7)	70 (2.48)	<0.001
23	. . . bought or sold anything you knew, or thought was stolen?	Never | *Once, 2 to 5 times, More than 5 times*	416 (15.1)	174 (12.5)	242 (17.8)	67 (2.37)	<0.001
24	. . .threatened or forced someone to give you money, a cell phone or similar?	Never | *Once, 2 to 5 times, More than 5 times*	118 (4.29)	54 (3.87)	64 (4.73)	74 (2.62)	0.265
25	. . .on purpose hit anyone hard enough to cause an injury or bleeding?	Never | *Once, 2 to 5 times, More than 5 times*	845 (30.7)	414 (29.7)	431 (31.7)	67 (2.37)	0.257
	*Sexuality*						
26	During your last sexual intercourse, did you use any contraception?	Condom, Birth control pill, Contraceptive implant, Other, Emergency contraception, Not yet had their first sexual intercourse | *No contraception was used*	258 (9.62)	89 (6.57)	169 (12.7)	140 (4.96)	<0.001
	**Safety**						
	Do you feel safe. . .						
27	. . .on your way to and from school?	Often or always, Sometimes | *Seldom or never*	44 (1.62)	21 (1.54)	23 (1.70)	103 (3.65)	0.735
28	. . . in the classroom?	Often or always, Sometimes | *Seldom or never*	54 (1.99)	26 (1.91)	28 (2.07)	105 (3.72)	0.762
29	. . . in school between classes?	Often or always, Sometimes | *Seldom or never*	58 (2.14)	34 (2.50)	24 (1.78)	110 (3.90)	0.195
30	. . .during daytime, outdoors in your neighbourhood?	Often or always, Sometimes | *Seldom or never*	51 (1.87)	24 (1.75)	27 (1.99)	97 (3.44)	0.637
31	. . .during night-time, outdoors in your neighbourhood?	Often or always, Sometimes | *Seldom or never*	96 (3.54)	51 (3.74)	45 (3.33)	108 (3.83)	0.560
32	. . . at home?	Often or always, Sometimes | *Seldom or never*	35 (1.29)	16 (1.18)	19 (1.40)	108 (3.83)	0.602

a Underlined words are used as contracted names of the questions in Table IV.

b The responses are dichotomised; the cut-off is marked with a vertical bar and the actual response options are in italics.

c All students=Y9+Y2U.

d Correspond to affirmative reports of the response options in italics.

e Differences in proportions between Y9 and Y2U, calculated by Pearson’s chi-square.

**Table III.** Dichotomised frequencies and proportions of symptoms, negative experiences and health-compromising behaviours reported in the 2014 survey. Items 2–5, 7–11, 15 and 18–32 were included in the confirmatory analysis.

No.	The items, verbatim translated from the questionnaire^a^	All selectable response options^b^	All students,^c^ *n* (%)^d^	Y9, *n* (%)^d^	Y2U, *n* (%)^d^	Missing,*n* (%)	Difference, Y9–Y2U, *p*-value^e^
	Male students		2358 (100)	1139 (48.3)	1219 (51.7)	-	-
	**School**						
1	Do you enjoy school?	A great deal, Quite a bit, Somewhat | *A little bit, Not at all*	100 (4.33)	61 (5.52)	39 (3.24)	47 (1.99)	0.007
2	Do you regularly skip class?	No, Once a semester, Once a month, 2 to 3 times a month| *Once a week, Twice a week or more*	108 (4.69)	53 (4.77)	55 (4.61)	55 (2.33)	0.859
	**Spare time**						
3	Are you satisfied with your spare time?	Very satisfied, Rather satisfied, Neither satisfied nor unsatisfied | *Rather unsatisfied, Very unsatisfied*	83 (3.60)	37 (3.33)	46 (3.85)	54 (2.29)	0.504
4	In your spare time, how many times a week do you exercise at least 30 minutes vigorously enough to make you sweat?	Every day, 4 to 6 times a week, 2 to 3 times a week, Once a week | *1 to 3 times a month, Less than once a month, Never*	536 (23.4)	255 (23.2)	281 (23.6)	65 (2.76)	0.833
	**Somatic health**						
5	How is your general health?	Excellent, Good, Fair | *Poor, Very poor*	69 (2.96)	32 (2.84)	37 (3.08)	30 (1.27)	0.731
6	How often do you brush your teeth in an ordinary day?	3 times a day or more, 2 times a day | *Once a day, Never*	496 (21.7)	211 (19.2)	285 (23.9)	70 (2.97)	0.006
	During the last three months, how often have you had. . .						
7	. . .headache (not counting migraine)?	Seldom or never, Once a month, Once a week | *More than once a week, Almost every day*	184 (8.23)	98 (9.15)	86 (7.38)	121 (5.13)	0.127
8	. . .stomach ache?	Seldom or never, Once a month, Once a week | *More than once a week, Almost every day*	135 (6.10)	67 (6.30)	68 (5.91)	144 (6.11)	0.706
9	. . .pain in your neck or shoulders?	Seldom or never, Once a month, Once a week | *More than once a week, Almost every day*	269 (12.2)	126 (12.0)	143 (12.4)	148 (6.28)	0.777
10	. . .pain in your back or hips?	Seldom or never, Once a month, Once a week | *More than once a week, Almost every day*	272 (12.3)	134 (12.6)	138 (12.0)	146 (6.19)	0.659
	**Mental health**						
11	Do you enjoy life right now?	A great deal, Quite a bit | *A little bit, Not at all*	176 (7.80)	81 (7.44)	95 (8.13)	101 (4.28)	0.538
	During the last three months, how often . . .						
12	. . . have you been concerned or worried?	Seldom or never, Once a month, Once a week | *More than once a week, Almost every day*	363 (16.3)	158 (14.7)	205 (17.8)	135 (5.73)	0.050
13	. . . have you experienced low mood?	Seldom or never, Once a month, Once a week | *More than once a week, Almost every day*	352 (15.9)	156 (14.6)	196 (17.0)	139 (5.89)	0.123
14	. . . have you been stressed?	Seldom or never, Once a month, Once a week | *More than once a week, Almost every day*	938 (41.5)	438 (40.2)	500 (42.8)	99 (4.20)	0.212
15	. . .did you feel cheerful?	*Seldom or never, Once a month, Once a week* | More than once a week, Almost every day	217 (9.46)	97 (8.81)	120 (10.1)	63 (2.67)	0.310
16	. . . did you have difficulties getting into sleep?	Seldom or never, Once a month, Once a week | *More than once a week, Almost every day*	624 (27.8)	291 (26.8)	333 (28.6)	111 (4.71)	0.344
17	During the last 12 months, how often have you tried to cut or injure yourself in any way?	Never | *Once, 2 to 5 times, More than 5 times*	135 (5.85)	73 (6.59)	62 (5.17)	51 (2.16)	0.144
	**Substance use**						
18	Do you smoke?	No, Have tried, Have stopped | *Sometimes, Daily*	384 (16.5)	103 (9.20)	281 (23.4)	37 (1.57)	<0.001
19	During the last 12 months, how often have you been drinking alcohol?	Never, Once, A few times every six months | *1 to 3 times a month, 1 to 2 times a week, More than 2 times a week*	561 (24.5)	105 (9.58)	456 (38.1)	66 (2.80)	<0.001
20	How many times have you ever used illegal drugs?	Never | *Once, Twice or more*	338 (14.7)	86 (7.84)	252 (20.9)	56 (2.37)	<0.001
	**Delinquency**						
	Have you ever. . .						
21	. . .taken goods from a shop without paying?	Never, Once, 2 to 5 times | *More than 5 times*	207 (9.30)	94 (8.85)	113 (9.70)	131 (5.56)	0.491
22	. . .broken into a house or a car?	Never | *Once, 2 to 5 times, More than 5 times*	286 (12.9)	118 (11.1)	168 (14.5)	141 (5.98)	0.016
23	. . . bought or sold anything you knew, or thought was stolen?	Never | *Once, 2 to 5 times, More than 5 times*	241 (11.0)	78 (7.51)	163 (14.1)	163 (6.91)	<0.001
24	. . .threatened or forced someone to give you money, a cell phone or similar?	Never | *Once, 2 to 5 times, More than 5 times*	72 (3.25)	35 (3.31)	37 (3.19)	140 (5.94)	0.881
25	. . .on purpose hit anyone hard enough to cause an injury or bleeding?	Never | *Once, 2 to 5 times, More than 5 times*	604 (27.3)	260 (24.6)	344 (29.8)	147 (6.23)	0.007
	**Sexuality**						
26	During your last sexual intercourse, did you use any contraception?	Condom, Birth control pill, Contraceptive implant, Other, Emergency contraception, Not yet had their first sexual intercourse | *No contraception was used*	209 (9.44)	62 (5.85)	147 (12.7)	143 (6.06)	<0.001
	**Safety**						
	Do you feel safe. . .						
27	. . .on your way to and from school?	Often or always, Sometimes | *Seldom or never*	36 (1.58)	16 (1.46)	20 (1.69)	83 (3.52)	0.663
28	. . . in the classroom?	Often or always, Sometimes | *Seldom or never*	38 (1.70)	23 (2.18)	15 (1.27)	120 (5.09)	0.097
29	. . . in school between classes?	Often or always, Sometimes | *Seldom or never*	46 (2.04)	27 (2.51)	19 (1.61)	102 (4.33)	0.130
30	. . .during daytime, outdoors in your neighbourhood?	Often or always, Sometimes | *Seldom or never*	45 (1.97)	24 (2.19)	21 (1.76)	74 (3.14)	0.461
31	. . .during night-time, outdoors in your neighbourhood?	Often or always, Sometimes | *Seldom or never*	73 (3.21)	34 (3.11)	39 (3.30)	82 (3.48)	0.801
32	. . . at home?	Often or always, Sometimes | *Seldom or never*	31 (1.38)	11 (1.03)	20 (1.69)	106 (4.50)	0.183

a Underlined words are used as contracted names of the questions in Table V.

b The responses are dichotomised; the cut-off is marked with a vertical bar and the actual response options are in italics.

c All students=Y9+Y2U.

d Correspond to affirmative reports of the response options in italics.

e Differences in proportions between Y9 and Y2U, calculated by Pearson’s chi-square.

### Missing data

The degree of missingness on each item was low (Tables II and III), but total missingness was 28% (2011) and 30% (2014) when using complete cases. Twenty data sets were therefore created in each survey by multiple imputation by chained equations [22].

### The exploratory factor analysis

EFAs were performed independently in the full sample (*N*=2823), in the Y9 data (*n*=1437) and in the Y2U data (*n*=1386) using polychoric correlations, the extraction method weighted least squares – mean and variance adjusted (WLSMV) and the oblique rotation geomin [23,24]. To determine the number of factors, a scree plot and Kaiser’s criterion were computed for each imputed data set [24,25] (Supplemental Figure S3).

The model was reanalysed until no cross-loadings and no weak factors appeared [26]. An item was kept in the model if it clearly loaded only onto one factor, that is, if one factor loading was at least twice as high as any other factor loadings on that item [26]. Smaller differences were taken to indicate cross-loadings; those items were deleted unless theoretically important [21,26]. Factors with fewer than three items were deemed as weak, while strong factors had at least five items with factor loadings of ⩾0.50 [25]. The meaning and naming of factors were discussed until the authors achieved consensus (Table IV).

**Table IV.** Exploratory factor analysis of poor mental-health symptoms and health-compromising behaviours in Swedish adolescent males in Y9 and Y2U using data from the 2011 survey.

No.^a^	Item^b^	Full model (all students, *N*=2823)				Y9 model (*n*=1437)				Y2U model (*n*=1386)			
		Deviancy	Unsafety	Gloominess	Pain	Deviancy	Unsafety	Gloominess	Pain	Deviancy	Unsafety	Gloominess	Pain
22	Broken into a house or a car	0.83				0.81				0.89			
18	Smoking	0.80				0.84				0.74			
24	Threatened someone	0.79				0.85				0.81			
20	Illegal drugs	0.77				0.79				0.74			
23	Bought anything stolen	0.76				0.76				0.80			
21	Taken goods without paying	0.71				0.72				0.77			
19	Drinking alcohol	0.71				0.73				0.64			
25	Hit anyone	0.65				0.67				0.69			
26	No contraception^c^	0.62				0.63				0.53			
2	Skipping class	0.54				0.58				0.49			
30	Unsafe outdoors, daytime^c^		0.95				0.95				0.96		
29	Unsafe between classes^c^		0.93				0.87				0.92		
28	Unsafe in the classroom^c^		0.92				0.86				0.91		
27	Unsafe to and from school^c^		0.89				0.87				0.89		
31	Unsafe outdoors, night-time^c^		0.87				0.86				0.88		
32	Unsafe at home^c^		0.84				0.84				0.80		
11	Do not enjoy life^c^			0.82				0.87				0.83	
5	Poor general health^c^			0.73				0.76				0.76	
3	Dissatisfied with spare time^c^			0.73				0.70				0.77	
15	Do not feel cheerful^c^			0.57				0.58				0.63	
4	Do not exercise^c^			0.30								0.26	
1	Do not enjoy school^c^							0.51				0.48	
10	Back pain				0.81				0.81				0.80
9	Neck pain				0.80				0.76				0.82
7	Headache				0.48			0.23^d^	0.39				0.56
8	Stomach ache				0.46			0.27^d^	0.41				0.49
Correlations:	Unsafety	0.03				0.32				0.07			
	Gloominess	0.09	0.34			0.12	0.14			−0.01	0.51		
	Pain	0.02	0.26	0.49		0.13	0.01	0.46		0.21	0.60	0.61	
Model fit:	Chi-square test (df)	2107 (206)				1043 (206)				1174 (227)			
	RMSEA		0.06				0.05				0.06		
	CFI		0.94				0.95				0.94		
	TLI		0.92				0.93				0.92		

The four factors deviancy, unsafety, gloominess and pain are here presented with standardised factor loadings, correlations between factors and model fit. Only factor loadings at least twice as large as the second largest loading are included.

a Number corresponding to the verbatim translation of the item in Table II.

b Contracted names of the items.

c The question was inverted, so a higher loading corresponds to an unhealthier situation.

d Cross-loading items.

RMSEA: root mean square error of approximation; CFI: comparative fit index; TLI: Tucker Lewis index.

### The confirmatory factor analysis

The models were validated in the imputed data sets from the 2014 survey using CFA based on polychoric correlations with WLSMV estimation [23]. First, the full model was tested in the full sample (*N*=2358), in Y9 only (*n*=1139) and in Y2U only (*n*=1219;Table V). Second, the Y9 model was tested in Y9 data (*n*=1139), and the Y2U model in Y2U data (*n*=1219). Third, the full model was tested in complete cases (*n*=1650) using diagonally weighted least squares estimation. Fourth, differences between school years were analysed using a multiple indicators and multiple causes (MIMIC) model based on the full model.

**Table V.** Confirmatory factor analysis of poor mental-health symptoms and health-compromising behaviours in Swedish adolescent males.

No.^a^	Item^b^	Full model in all students (*N*=2358)^c^				Full model in Y9 (*n*=1139)^c^				Full model in Y2U (*n*=1219)^c^			
		Deviancy	Unsafety	Gloominess	Pain	Deviancy	Unsafety	Gloominess	Pain	Deviancy	Unsafety	Gloominess	Pain
22	Broken into a house or a car	0.82				0.84				0.82			
18	Smoking	0.77				0.75				0.73			
24	Threatened someone	0.89				0.97				0.82			
20	Illegal drugs	0.79				0.83				0.75			
23	Bought anything stolen	0.82				0.89				0.77			
21	Taken goods without paying	0.68				0.69				0.71			
19	Drinking alcohol	0.68				0.70				0.59			
25	Hit anyone	0.66				0.72				0.63			
26	No contraception^d^	0.58				0.59				0.50			
2	Skipping class	0.53				0.58				0.47			
30	Unsafe outdoors, daytime^d^		0.95				0.94				0.96		
29	Unsafe between classes^d^		0.96				0.96				0.94		
28	Unsafe in the classroom^d^		0.96				0.97				0.96		
27	Unsafe to and from school^d^		0.93				0.94				0.91		
31	Unsafe outdoors, night-time^d^		0.82				0.80				0.84		
32	Unsafe at home^d^		0.92				0.93				0.92		
11	Do not enjoy life^d^			0.90				0.90				0.91	
5	Poor general health^d^			0.80				0.78				0.82	
3	Dissatisfied with spare time^d^			0.64				0.58				0.70	
15	Do not feel cheerful^d^			0.70				0.72				0.69	
4	Do not exercise^d^			0.19				0.16				0.21	
10	Back pain				0.78				0.78				0.78
9	Neck pain				0.78				0.79				0.76
7	Headache				0.56				0.58				0.55
8	Stomach ache				0.60				0.58				0.63
Correlations:	Unsafety	0.17				0.28				0.11			
	Gloominess	0.18	0.35			0.22	0.33			0.12	0.39		
	Pain	0.20	0.23	0.39		0.23	0.20	0.38		0.20	0.25	0.42	
Model fit:	Chi-square test (df)	2288 (269)				1255 (269)				1109 (269)			
	RMSEA	0.06				0.06				0.05			
	CFI	0.95				0.95				0.95			
	TLI	0.94				0.94				0.94			

The full model from the EFA (Table IV) was confirmed in 2014 survey data from all students, from only Y9 and from only Y2U, and is here presented with standardised factor loadings, correlations between factors and model fit.

a Number corresponding to the verbatim translation of the item in Table III.

b Contracted names of the items.

c Standardised factor loadings.

d The question was inverted, so a higher loading corresponds to an unhealthier situation.

### Fit indices and software

Model fit was assessed by root mean square error of approximation (RMSEA), comparative fit index (CFI) and Tucker–Lewis index (TLI). For RMSEA, values <0.05 suggest good fit, 0.05–0.08 acceptable fit, 0.08–0.10 marginal fit and >0.10 poor fit [24]. For CFI and TLI, values >0.95 [27], or >0.90 in large data sets [21], suggest good model fit.

R v3.5.1 in RStudio v1.1.463 with package psych, mice and lavaan (R Foundation for Statistical Computing, Vienna, Austria) and Mplus 8 (Muthén & Muthén, Los Angeles, CA) were used.

## Results

### Healthy males with unhealthy behaviours

In both surveys (2011 and 2014), the studied population consisted of 15- to 16-year-olds (Y9) and 17- to 18-year-olds (Y2U) in equal proportions (Table I). The majority reported high well-being and good physical health without chronic conditions or somatic symptoms (Tables I–III). In general, they enjoyed their spare time, fulfilled school and felt secure in everyday life. Nevertheless, one quarter consumed alcohol every month, one sixth smoked regularly and one eighth had used illegal drugs or committed burglary. Substance use and delinquency were, however, more frequently reported among the older adolescent males than among their younger peers (Tables II and III).

### EFA

The scree plot indicated three factors (Supplemental Figure S3). In 14/20 data sets, Kaiser’s criterion suggested three factors; in the remaining six data sets, it suggested four factors. Models with three, four and five factors were developed independently in the full sample and in the subsets (Y9 and Y2U). In all three samples, the five-factor models degenerated into four-factor models due to factor weakness. All four-factor models had better model fit than the corresponding three-factor model, and therefore the four-factor model was considered superior.

#### The final four-factor model in the full sample

The final model consisted of four factors: deviancy, unsafety, gloominess and pain. In the full sample, the model contained 25 items. Model fit was acceptable to good: RMSEA=0.06, CFI=0.94 and TLI=0.92 (Table IV). Deviancy (factor loadings 0.54–0.83) comprised 10 items about substance use, delinquency and non-use of contraceptives. Unsafety (factor loadings 0.84–0.95) comprised six items about feelings of unsafety in the neighbourhood, in school and at home, day and night, indoors and outdoors, with peers and with adults. Gloominess (factor loadings 0.30–0.82) comprised five items about dissatisfaction with life and spare time, poor general health, lack of cheerfulness and exercise. Pain (factor loadings 0.46–0.81) comprised four items: pain in the back, neck, head and stomach. Gloominess correlated moderately with both pain and unsafety. Seven items were deleted due to cross-loadings (Table II: items 1, 6, 12–14, 16 and 17). The items about worry, low mood, stress and insomnia loaded on gloominess and pain, indicating a relation to both. Similarly, not enjoying school loaded on gloominess and unsafety, whereas habitually not brushing teeth and a history of self-injury loaded on gloominess, unsafety and deviancy.

#### Model differences due to age

The Y9 and Y2U models had acceptable to good model fit and contained the same four factors as the full model, with minor differences at item level in gloominess and in factor correlations (Table IV). Compared to the Y9 model, unsafety correlated more strongly with gloominess and pain in the Y2U model, but less strongly with deviancy. In the Y9 model, headache and stomach ache had cross-loadings, but these items had to be kept to avoid deletion of pain. All item deletions in both subsets were hence based on the same cross-loadings as in the full model.

### Good model fit in CFA

The full model was confirmed in the 2014 data with acceptable to good model fit in the full sample, in the Y9 subset and in the Y2U subset using imputed data (Table V), as well as in the full sample with complete cases only (*n*=1650; RMSEA=0.05, CFI=0.97 and TLI=0.97). The Y9 model had acceptable to good fit in the Y9 subset (RMSEA=0.06, CFI=0.95, TLI=0.95), as had the Y2U model in the Y2U subset (RMSEA=0.05, CFI=0.95, TLI=0.94). In all six CFAs, gloominess correlated moderately to pain (ρ=0.38–0.43) and to unsafety (ρ=0.33–0.43), equally to the full EFA model. Moreover, the pattern of factor loadings was basically consistent throughout all EFA and CFA models.

Nonetheless, the MIMIC analysis revealed that the average deviancy and gloominess were 0.536 SD (*p*<0.001) and 0.150 SD (*p*=0.002) higher, respectively, in Y2U than in Y9, whereas the average unsafety was 0.154 SD (*p*=0.018) lower. The average pain did not differ between the school years (*p*=0.201).

## Discussion

The present factor model of poor mental-health symptoms, somatic symptoms and HCBs in adolescent males contained four underlying factors: deviancy, unsafety, gloominess and pain. The model arguably offers theoretical guidance when addressing these interrelated and essential topics in clinic or in research. In some respects, our findings are original. Deviancy and gloominess have previously been described in the literature, but to the best of our knowledge, unsafety and pain have not been seen in a similar model before.

In our final model (i.e. the full model; Tables IV and V), the strong factor deviancy aggregated several socially deviant behaviours and was therefore interpreted as an inclination to deviate from the norm. When excessive or continual, deviancy may stifle health or social development, whereas in most adolescents, its manifestations can be thought of as normal exploration, leading to experience and thus more informed future decisions [9,11]. The factor is congruent with *The Problem Behaviour Theory* from 1991, a large Brazilian study from 2016 and a still larger American study from 2019 [10,11,13,15]. In other studies, the behaviours in deviancy are accounted for by two or three factors. However, differences in method, item selection and area of interest may explain this discrepancy [8,12]. In sum, the theory of a general underlying factor of deviant behaviour may still be accurate among the world’s adolescent males.

Unsafety, which aggregated feelings of unsafety in a wide range of locations and circumstances, was interpreted as a general tendency to feel unsafe. This strong factor is a little like a previously described factor containing being afraid, worry and insomnia [28], but in the present study, worry and insomnia were affected not by unsafety but by gloominess and pain. Unsafety does, however, bear resemblance to the personality trait ‘social inhibition’, that is, an inclination to feel insecure in social activities [29]. Another possible explanation is that unsafety reflects intimidating experiences, such as harassment, bullying or violence. If the general inclination to feel unsafe includes medical consultations, the unsafety factor may have important clinical implications.

Gloominess, which reflects a tendency for a general lack of well-being and a negative outlook, has been described previously [28]. Gloominess also bears resemblance to the personality trait ‘negative affectivity’, a vulnerability for dysphoria and anxiety [29]. In the present study, most of the dysphoric and anxious symptoms (Table II, items 12–14 and 16) loaded equally on gloominess and pain. This finding agrees with known associations between negative affectivity and pain conditions in adolescents [30] in which worry has been suggested as a central link [7]. It also indicates that either gloominess or pain can be expressed as dysphoria and anxiety.

Pain included painful bodily sensations from several tissue types and organs. An organic disorder was therefore considered a less likely explanation than a general tendency to express unpleasant emotions in terms of pain.

### Gloominess correlated with unsafety and pain but not with deviancy

The moderate correlation between gloominess and pain agrees with earlier findings of concordant depression and pain conditions in adolescent males [6,30]. In fact, gloominess and pain might be two aspects of the same factor, as indicated by the cross-loadings in the Y9 subset. Still, in the present study, such a three-factor model had a poorer model fit than the four-factor model, contradicting the idea of a common gloominess and pain factor. Altogether, even though the results are a bit ambiguous, in this specific sample, pain appears to be an independent factor, at least among older adolescent males.

Despite known associations between poor mental health and HCBs [8], gloominess and deviancy correlated only weakly in our final model. A possible explanation is that the studied adolescent males were in, or recently past, the exploratory phase of normal development, in which a moderate impact of deviancy is healthy and normal [9,11].

### Deviancy increased with age

Age did not affect the model structure. Yet, in congruence with previous studies, the average level of deviancy increased with age [10,14], reflecting a higher proportion of ongoing or passed exploration. Theories on normal development may also explain that gloominess was slightly more and unsafety slightly less pronounced among older adolescent males, who generally have a firmer sense of identity and a more realistic, and sometimes pessimistic, outlook on life [2].

### Implications

The present model has potential implications in the clinic, in research and in the field of public health. By verbally exploring the four areas, deviancy, unsafety, gloominess and pain, health-care workers can obtain theoretical guidance in assessing adolescent males’ health risks. The model can also be used in research of any phenomenon associated with the health of adolescent males, when the goal is a conceptual understanding. Furthermore, the correlations between unsafety, gloominess and pain imply that it may be advantageous to include these three perspectives in interventions against poor mental health.

### Strengths and limitations

A strength of the study was the large study size. Another strength was the study design, with two analogous populations in a region socio-economically representative of Sweden as a whole. The model can thus be generalised to Swedish adolescent males and, with some caution, to other similar populations.

A third strength was the use of factor analysis. The data were explored without any assumptions beyond that the covariation is caused by underlying factors, which enabled findings of new associations and a general conceptual understanding.

Some limitations need to be mentioned. First, the questionnaires were not designed for factor analysis. The most appropriate items were selected from a pre-existing questionnaire, and interesting aspects may have been lost. Still, the present model gives, if not a comprehensive, at least a broad picture.

A second limitation is the use of a school-based survey. Non-participating adolescent males may have skipped school more frequently than the study population, which gives a selection bias towards students with lower levels of truancy and other HCBs. However, the impact on the model is diminished by the study’s high participation rate.

Third, the clustering effect of schools was not considered, since the participants’ anonymity made data about school enrolment unavailable. The precision of the study might be overestimated but is unlikely to affect the model structure.

A fourth limitation is that the study was not designed to analyse what background variables influence the expression of the described factors, such as negative experiences, circumstances at home and in school, relationship with parents and peers, as well as socio-economics and genetics. Future studies analysing these associations would be most welcome.

Finally, it should be noted that the model is based upon a written questionnaire, and future studies need to investigate its applicability in medical face-to-face consultations. The usefulness of the model would also benefit from validating studies in other populations.

## Conclusions

Poor mental health and HCBs constitute important, interrelated and partly preventable aspects in adolescent males’ health. Whenever a comprehensive approach is needed in public-health matters, in medical consultations or in research, this factor model may provide theoretical guidance. Its four areas, deviancy, unsafety, gloominess and pain, may lend support to intervention programmes, health-care workers’ assessments or researchers’ hypotheses.

## Supplemental Material

sj-pdf-1-sjp-10.1177_1403494820974555 – Supplemental material for Understanding adolescent males’ poor mental health and health-compromising behaviours: A factor analysis model on Swedish school-based data Click here for additional data file. Supplemental material, sj-pdf-1-sjp-10.1177_1403494820974555 for Understanding adolescent males’ poor mental health and health-compromising behaviours: A factor analysis model on Swedish school-based data by Johanna Haraldsson, Ronnie Pingel, Lena Nordgren, Ylva Tindberg and Per Kristiansson in Scandinavian Journal of Public Health
