# Supplementary material for: Understanding adolescent males’ poor mental health and health-compromising behaviours: A factor analysis model on Swedish school-based data
Source: Scand J Public Health. 2020 Dec 15;50(2):232–44. doi: 10.1177/1403494820974555 (PMC8873304; doi:10.1177/1403494820974555)

Understanding adolescent males' poor mental health and health-compromising behaviours — a factor analysis model on Swedish school-based data

Haraldsson J, Pingel R, Nordgren L, Tindberg Y, Kristiansson P.

Supplemental Figure 1: The recruitment of the study population in the survey *Life and Health in Youth* 2011.

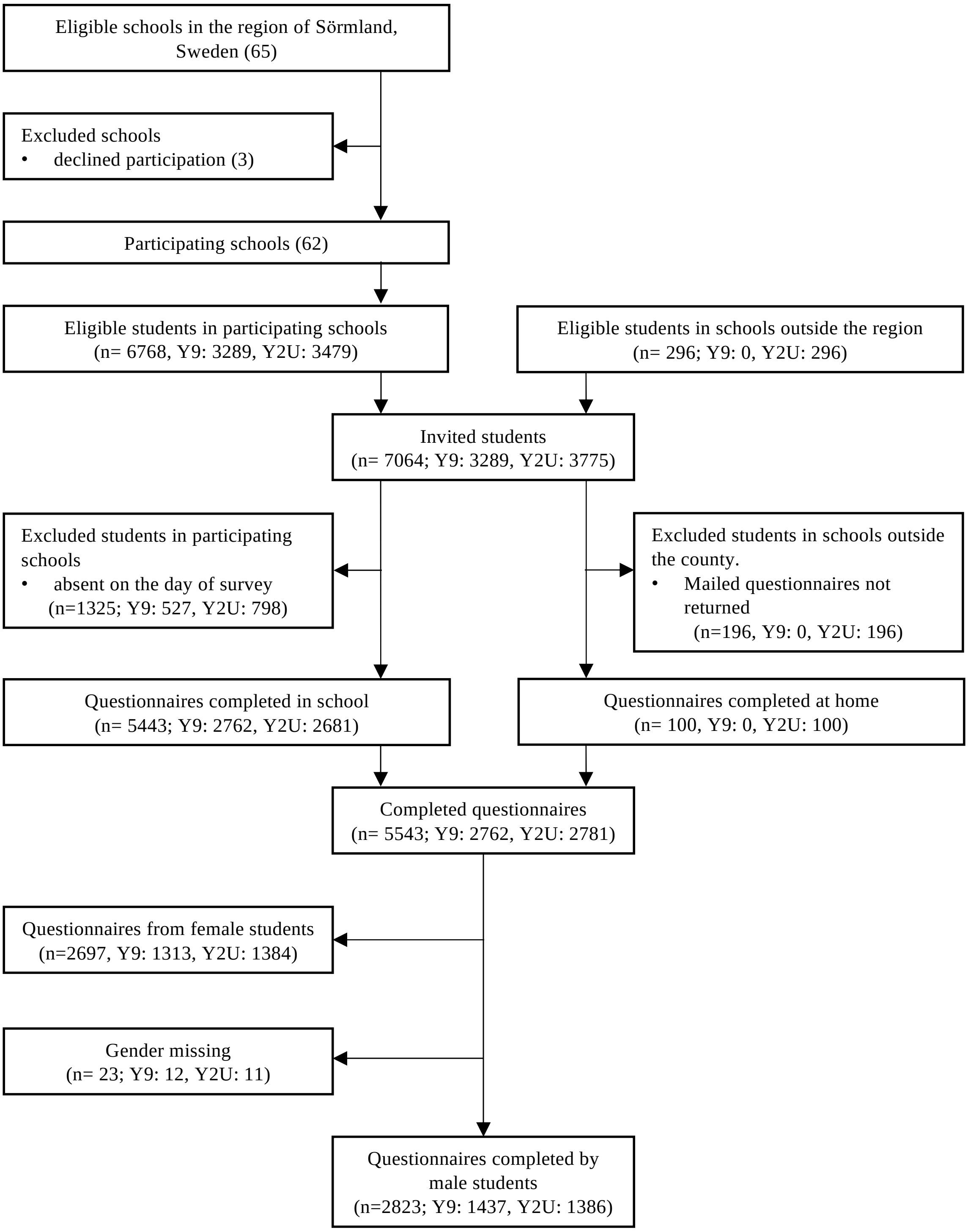

Supplemental Figure 2: The recruitment of the study population in the survey Life and Health in Youth 2014

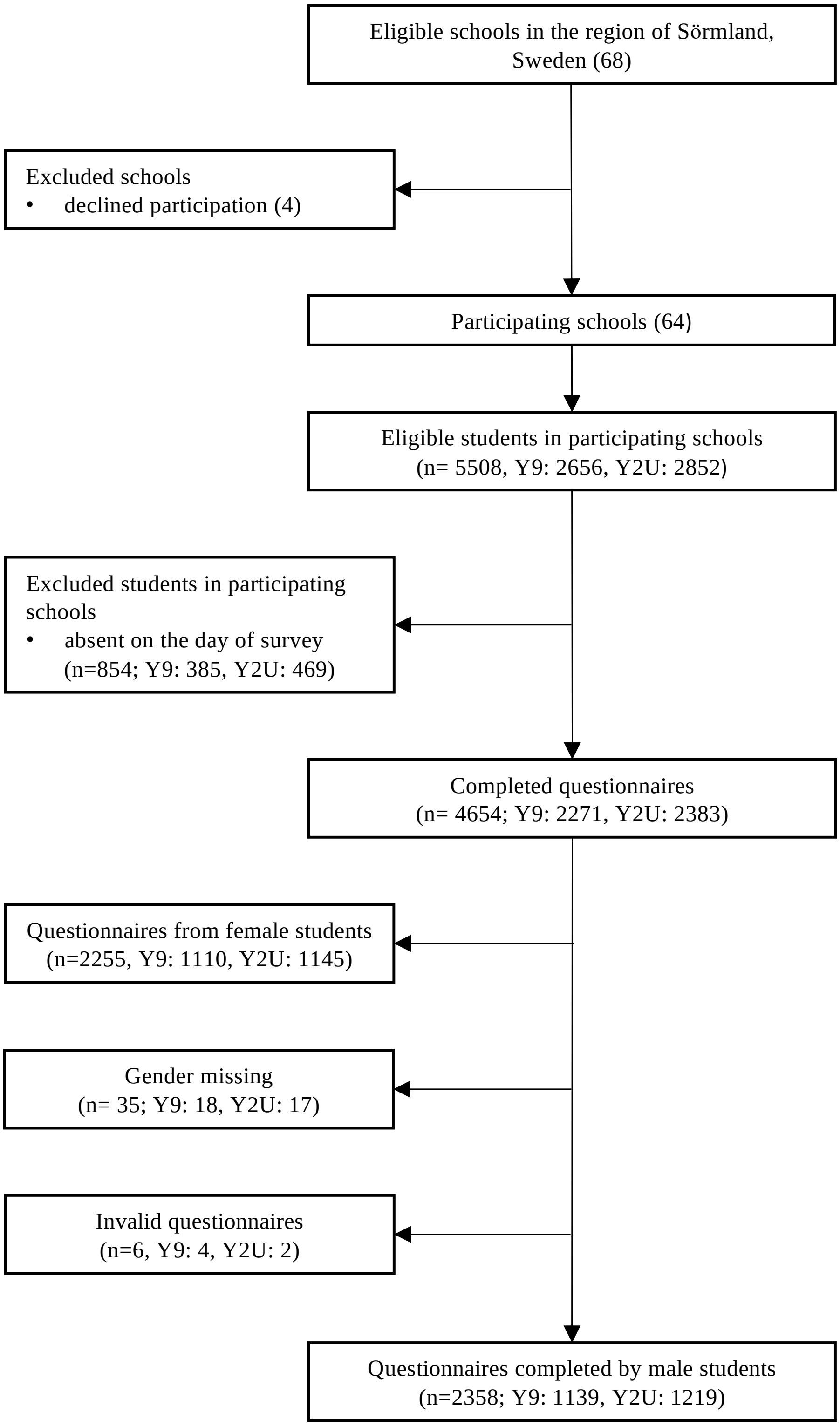

Supplemental Figure 3: The scree plot for the total dataset (n=2823) in the 2011 survey, based on data from twenty imputed datasets.

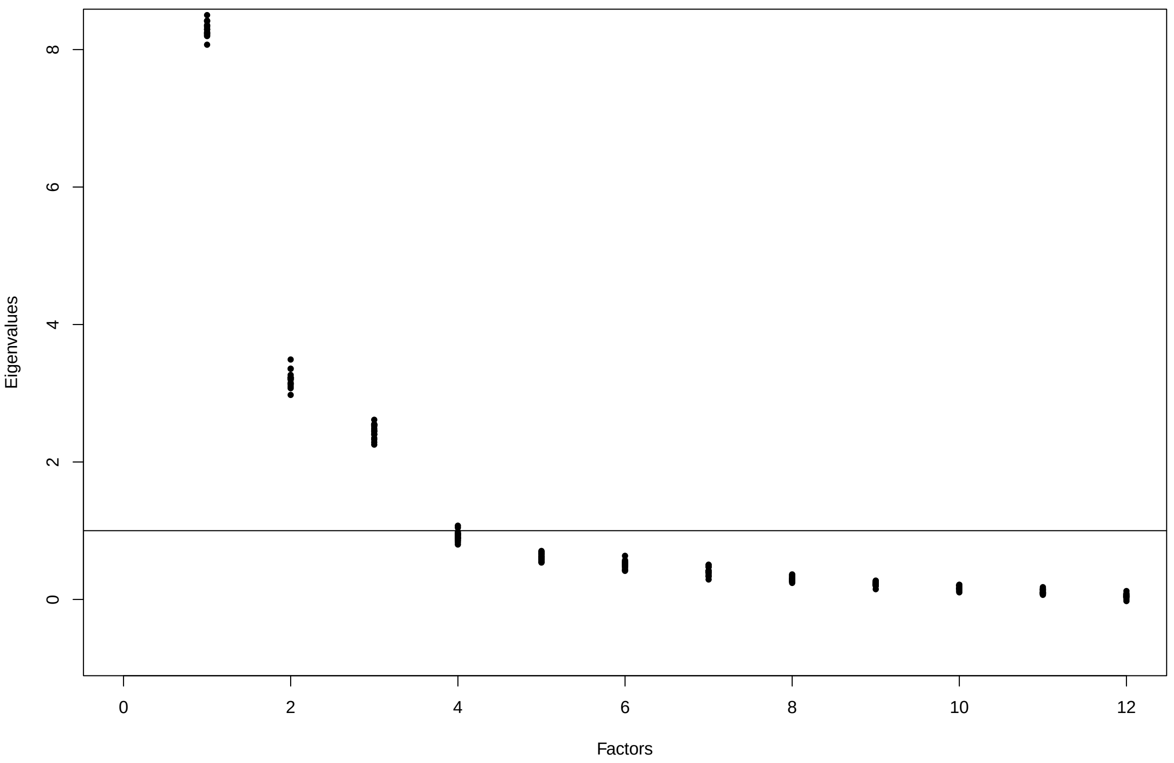

Supplement: sj-pdf-1-sjp-10.1177_1403494820974555 – Supplemental material for Understanding adolescent males’ poor mental health and health-compromising behaviours: A factor analysis model on Swedish school-based data [file sj-pdf-1-sjp-10.1177_1403494820974555.pdf]
